# Integrative analysis of the ovarian metabolome and transcriptome of the Yaoshan chicken and its improved hybrids

**DOI:** 10.3389/fgene.2024.1416283

**Published:** 2024-07-08

**Authors:** Xiaomeng Miao, Tian Wu, Hongyuan Pan, Yalan Zhang, Jia Liu, Ying Fan, Lin Du, Yu Gong, Liang Li, Tengda Huang, Zhonghua Ning

**Affiliations:** ^1^ Institute of Animal Husbandry and Veterinary Medicine, Guizhou Academy of Agricultural Sciences, Guiyang, China; ^2^ College of Animal Science and Technology, China Agricultural University, Beijing, China; ^3^ College of Animal Science and Technology, Guangxi University, Nanning, China; ^4^ Guizhou Province Livestock and Poultry Genetic Resources Management Station, Guiyang, China

**Keywords:** egg production, ovary, chicken, transcriptome, metabolomics

## Abstract

**Introduction:** Laying performance is a key factor affecting production efficiency in poultry, but its molecular mechanism is still indistinct. In this study, Yaoshan chickens, a local breed in Guizhou, China, and merchant chickens (GYR) with higher egg yield after the three-line cross improvement hybridization of Yaoshan chickens were used as animal samples.

**Methods:** To explore the regulatory mechanism of the diversities in laying performance, RNA-seq and ultra-performance liquid chromatographytandem mass spectrometry (UPLC—MS/MS) were used to describe the transcriptional and metabolic profiles of the ovaries of Yaoshan and GYR chickens.

**Results:** At the transcriptional level, 288 differentially expressed genes were upregulated in Yaoshan chickens and 353 differentially expressed genes were upregulated in GYR chickens. In addition, GSEA showed that ECM-receptor interactions and the TGF-β signaling pathway were restrained, resulting in increased egg production in GYR chickens. Furthermore, the upregulation of thiamine and carnitine was identified by metabolomic analysis to facilitate the laying performance of hens. Finally, comprehensive analyses of the transcriptome and metabolome found that thiamine and carnitine were negatively correlated with ECM-receptor interactions and the TGF-β signaling pathway, which jointly regulate the laying performance of Yaoshan chickens and GYR chickens.

**Discussion:** Taken together, our research delineates differences in the transcriptional and metabolic profiles of the ovaries of Yaoshan and GYR chickens during the peak egg production period and provides new hypotheses and clues for further research on poultry egg production performance and the improvement of economic benefits.

## 1 Introduction

Yaoshan (YS) chickens, an indigenous chicken breed in China, are mainly produced in Guizhou Province. They are favoured by local consumers because of their bright feathers, rough feeding resistance, strong stress resistance and good quality meat and eggs; however, they also have shortcomings such as poor egg production performance. The laying performance includes factors such as age and body weight at first egg, egg quality and egg production, which are closely related to the profit that enterprises raising poultry can make (Liu et al., 2019; Du et al., 2020). Therefore, we utilized high-yielding recessive white feather layers (RW) and green-shell chickens with yellow feathers (GY) to crossbreed and improve Yaoshan chickens. Commercial chickens (GYR) with high reproductive capability were obtained by means of three-line hybridization [GY×(YS×RW)] and have the ideal appearance characteristics of shallow plumage colour, shallow shank colour and elegant body shape; moreover, egg production increased while the excellent meat and egg quality of Yaoshan chickens was maintained.

The ovary is the reproductive organ of female animals, which can ovulate and secrete a variety of reproductive hormones. The level of egg production in poultry is determined by the function of the ovary and regulated by the hypothalamic-pituitary-gonadal axis (HPG) (Zhang et al., 2019). Previous studies have shown that poultry with good egg production have a higher ovary index and thicker granulosa cell layer (Yang et al., 2019).

The transcriptome is the sum of all RNAs that can be transcribed in cells under specific physiological conditions, reflecting the regulation of genes at the transcriptional level (Xue et al., 2017). Currently, transcriptomics is widely used in the study of growth performance (Wang et al., 2021), meat quality (Li et al., 2020; Pan et al., 2022), reproductive performance (Cindrova-Davies et al., 2017), pathogenic mechanisms (Ni et al., 2019; Perlas et al., 2021) and other important aspects in poultry that may affect economic benefits. He et al. performed transcriptome analysis on the leg muscle tissue of Bian chickens and filtered genes related to the growth rate of muscle and the significant pathways (He et al., 2020). Yang et al. conducted a systematic analysis of transcriptomic data and found 10 candidate genes, such as FADS2, DCN and FRZB, that significantly affected the synthesis of polyunsaturated fatty acids in chickens, thus providing a direction to improve meat quality during chicken breeding (Piórkowska et al., 2016). Porter et al. revealed the production of steroid hormones during follicular development and the regulation of follicular development in high-yielding and low-yielding layers using transcriptome sequencing (Yang et al., 2020). Yang et al. found a series of genes closely related to the length of time that sperm are stored in the junction of the uterus and vagina of hens, which laid a theoretical foundation for further improving the fertilization rate of hens (Brady et al., 2021).

Metabolomics is a new discipline that simultaneously carries out qualitative and quantitative analyses on all low-molecular-weight metabolites of a certain organism or cell in a specific physiological period and can reflect the changes in genes or the environment in biological systems (Ren et al., 2018). According to the desired research purpose, metabolomics can be further divided into nontargeted and targeted approaches. Nontargeted metabolites can be used to find biomarkers more effectively (Naz et al., 2014). At present, metabonomics has been widely used in various fields of animal study. Ying et al. used LC‒MS to measure the metabolic components in the serum of geese raised with different densities, which provided unique insights into the mechanism of adverse effects on the health of geese raised under high-density conditions (Ying et al., 2021). Shi et al. combined transcriptome and metabolome analyses to analyse ascites syndrome, a disease that greatly affects the production efficiency of broilers, and came to valuable conclusions (Shi et al., 2014). Huang et al. found potential gene metabolite pairs affecting the reproduction of laying hens through combined analysis of the transcriptome and metabolome (Huang et al., 2022).

At present, there are no relevant studies on the transcriptome and metabolome of YS chicken ovarian tissue, which limits the utilization of YS chickens. In this study, we tracked the laying performance of YS and GYR hens. These animals were subjected to transcriptome and metabolome high-throughput sequencing to explore the molecular regulation of ovarian tissue in the context of differences in egg production. Therefore, this research could provide a reference for the utilization of excellent livestock and poultry breeds and research on poultry reproductive performance.

## 2 Materials and methods

### 2.1 Ethical statement

All experimental procedures and the collection of all tissue samples from YS and GYR chickens completely met the relevant standards on animal welfare established by the relevant guidelines set by the Ethics Committee of Guizhou Animal Husbandry and Veterinary Research Institute (JXYJS-20190312).

### 2.2 Animals and sample collection

Hybridization pattern of the experimental chicken (GYR) in this study: green-shell chickens with yellow feathers (GY: ♂) were hybridized with hens (RR: ♀) generated from the cross of Yaoshan chickens (YS: ♂) and the recessive white feather layers (RW: ♀).

**FIGURE 1:**
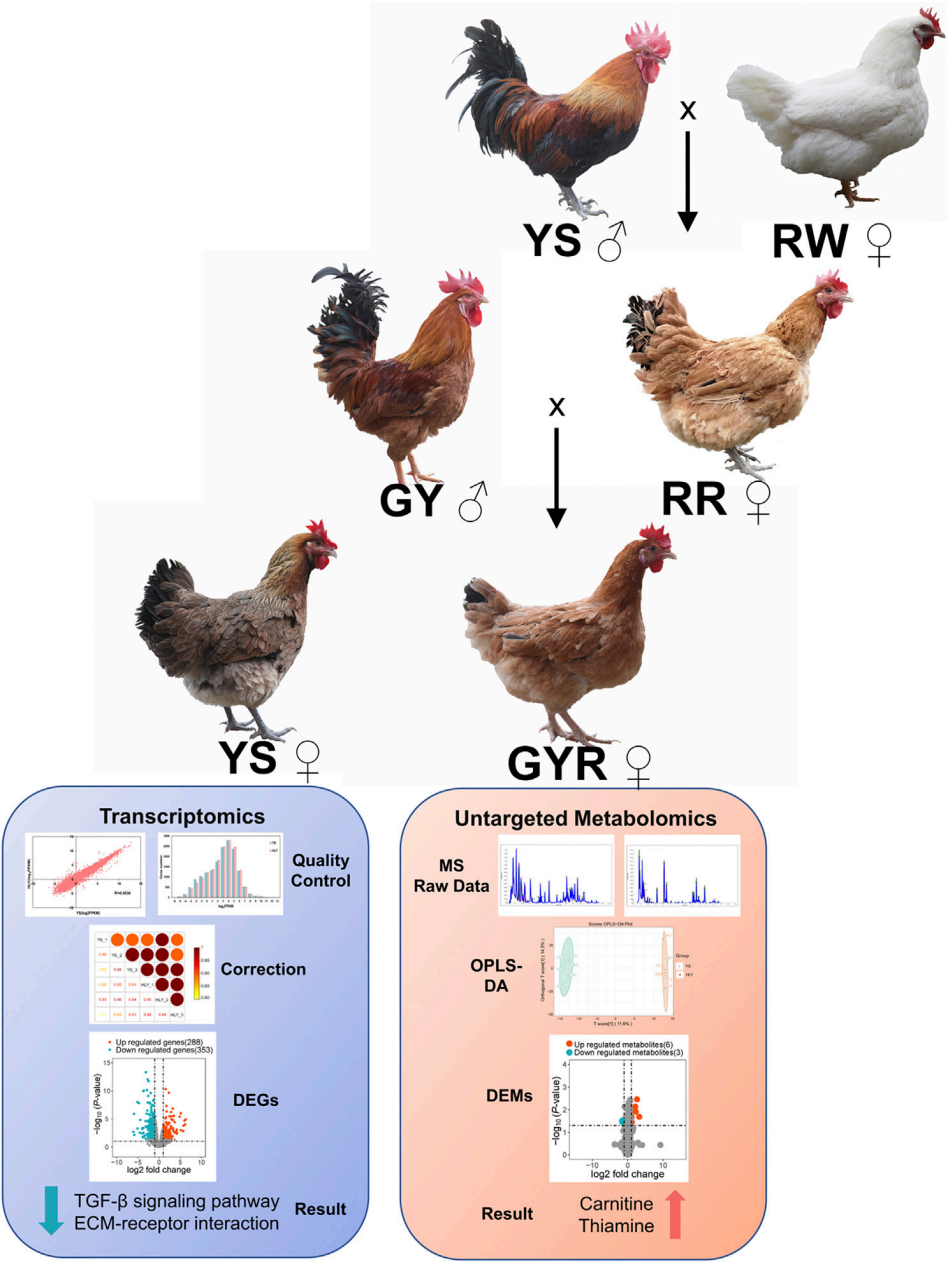
Clinical trial flow diagram. The diagram indicates selection process, the trial set-up, the collection of samples, and the analysis of transcriptome and metabolomics.

YS and GYR chickens are both hatched in the same batch, and the hen breeds and management mentioned in this pattern were provided in the breeding centre of Lvyuan Poultry Industry Co., Ltd. in Guiyang, China. The YS and GYR chickens, with 80 in each group, were fed in a controlled environment with free access to water and food. The nutritional level of the hens was the same (Table 1). Vaccinations followed the poultry vaccination guidelines established by the local animal husbandry and veterinary authorities. The age, body weight, egg number, egg weight and yolk weight were recorded for each chicken. At 260 days of age, 6 chickens that had produced average laying for the GYR and YS groups were randomly chosen and humanely sacrificed. The ovarian tissues with all the visible follicles for each chicken removed were rinsed with saline, quickly frozen in liquid nitrogen, and stored at −80°C, then used for omics sequencing (Figure 1).

**TABLE 1:** Nutrient levels of the basal diet.

Items	1–6 weeks	7–18 weeks	19–37 weeks
ME/(MJ/kg)	11.92	11.3	11.51
Crude protein/%	18	14	16.5
Crude fat/%	3	3	3
Crude fiber/%	3	3	3
Calcium/%	0.9	1	3.2
Available P/%	0.45	0.4	0.4
NaCl/%	0.36	0.36	0.32
Met + Cys/%	0.71	0.56	0.64

### 2.3 Transcriptome sequencing

TRIzol RNA extraction reagent was used to isolate total RNA from the YS and GYR groups. A total amount of 1 µg of RNA per sample was used as the input material for the RNA sample preparations. Transcriptome sequencing was performed by Metware Biotechnology Inc. (Wuhan, China). A NanoPhotometer spectrophotometer (IMPLEN, Westlake Village, CA, United States) was used to determine the purity of the RNA. A Qubit RNA Assay Kit in a Qubit 2.0 fluorometer (Life Technologies, CA, United States) was used to measure the concentration of RNA. The RNA Nano 6000 Assay Kit of the Bioanalyzer 2,100 system (Agilent Technologies, CA, United States) was used to assess the integrity of the RNA. After the RNA samples met the standard, mRNA was purified from total RNA using poly-T oligo attached magnetic beads. Divalent cations were used to carry out fragmentation in NEBNext First Strand Synthesis Reaction Buffer (5X) at high temperature. Random hexamer primers and M-MuLV Reverse Transcriptase (RNase H-) were used to synthesize first-strand cDNA. Subsequently, DNA polymerase I and RNase H were used to synthesize second-strand cDNA. After cDNA purification, end repair, dA‐tailing, adapter ligation and AMPure XP beads were used to screen approximately 200 bp of cDNA. Then, PCR amplification was performed, and AMPure XP beads were used again to purify the PCR products. After library construction, initial quantification was performed using a Qubit 2.0 fluorometer, and the library was diluted to 1.5 ng/μL. Afterwards, an Agilent 2,100 bioanalyzer was used to detect the insert size of the library, and qRT‒PCR was used to accurately quantify the effective concentration of the library (effective concentration >2 nM). According to the instructions of the manufacturer, clustering of the index-coded samples was performed on a cBot Cluster Generation System using TruSeq PE Cluster Kit v3-cBot-HS (Illumina). After cluster generation, an Illumina Nova 6,000 platform was used to sequence the library preparations, yielding paired-end reads of 125 bp/150 bp.

### 2.4 Transcriptome analysis

Convert Illumina high-throughput sequencing results to Raw Reads and store them in the FASTQ file format. Removal of low-quality sequences and linker contamination from Raw Reads by the fastp (version 0.19.3) platform for analysis of Clean Reads (Chen et al., 2018). HISAT2 v2.1.0 was used to construct the index and compare the reference genome with its annotation (GRCg7b.108) files downloaded from the designated website (Kim et al., 2019). The fragment per kilobase of transcript per million mapped reads (FPKM) of each gene was calculated to quantify its expression abundance. DESeq2 was used to identify the differential expressed genes (DEGs). The |log_2_ fold change| > 1 and *p*-value <0.05 were used as the thresholds of DEGs in the transcriptome.

### 2.5 Metabolomics sequencing

Standard tissue sample treatment was carried out and used for on-instrument analysis (Huang et al., 2022). The samples were cut, mixed, multi-point sampled, and weighed into 20 mg (± 1 mg) centrifuge tubes with corresponding numbers. The samples were centrifuged at 3,000 r/min for 30 s at 4°C to the bottom of the tubes. After the samples were centrifuged, 400 μL of internal standard extraction solution with 70% methanol water was added. After the samples were shaken at 1,500 r/min for 5 min, they were allowed to stand on ice for 15 min. Then centrifuge at 4°C at 12,000 rpm for 10 min. Transfer 300 μL of the supernatant into a centrifuge tube with a separate number and let it stand in a refrigerator at −20°C for 30 min. At 4°C, centrifuge at 12,000 r/min for 3 min and transfer 200 μL of the supernatant to the liner of the corresponding vial for on-machine analysis. Then, the filtrate was injected into the LC-MS/MS system for analysis. LC-MS/MS analysis was performed using ultra-performance liquid chromatography (ExionLC AD, https://sciex.com.cn/) and a four-stage time-of-flight mass spectrometer (TripleTOF 6600, AB SCIEX) by injecting experimental samples into an ACQUITY HSS T3 column (2.1 × 100 mm, 1.8 μm) with linear gradient time and flow rate settings of 16 min and 0.2 mL/min, respectively. Eluents A (0.1% FA, water) and B (methanol) were used for the positive pattern events. The negative mode customers are a (5 mM ammonium acetate, pH 9.0) and B (methanol). Gradient of solvent: 5% B, 0 min; 90% B, 10.0 min; 90% B, 11.0 min; 5% B, 11.1 min; 5% B, 14 min. The electrospray ionization mass spectrometer (ESI) spray voltages were set to 5,500 V (positive) and −4,500 V (negative), the temperature was set to 500°C, and the intrathecal gas flow rate was 0.35 mL/min.

### 2.6 Metabolomics analysis

Peaks were obtained by peak matching using Ultra Performance Liquid Chromatography (ExionLC AD, https://sciex.com.cn/) and Tandem mass spectrometry (QTRAP^®^, https://sciex.com/) and each metabolite was quantified from raw data files obtained by UPLC-MS/MS/MS. Accurate characterization was carried out based on the self-built target standard database MWDB (http://en.metware.cn/list/27.html, accessed 20 August 2020), mHK database (The databases information including Metlin, https://metlin.scripps.edu, accessed 20 August 2020; HMDB, https://hmdb.ca/, accessed 20 August 2020; KEGG, https://www.genome.jp/kegg/, accessed 20 August 2020) and MetDNA of Maiwei. Multi-ion pair information (http://metdna.zhulab.cn/) and the retention times (RTs) of the identified metabolites were extracted and combined with the target database built by MAVIR. Moreover, Q-Trap was used to accurately quantify the population samples.

To compare data of different orders of magnitude, the peak areas were normalized by batch normalization. Based on the metabolome characteristics, data were standardized prior to typing in samples. To obtain more reliable and intuitive results, the software automatically models the data fitting analysis. Positive crossover partial least squares discriminant analysis (OPLS-DA) was then used to supervise the data analysis, and permutation tests were used to prevent overfitting of the supervision model.

### 2.7 GO and KEGG enrichment analysis of DEGs

The program clusterProfiler R was used for Gene Ontology (GO) and Kyoto Encyclopedia of Genes and Genomes (KEGG) enrichment analysis of the DEGs and SDMs. The *p*-value < 0.05 was taken as a metric for quantifying the pathways and gene ontological processes.

### 2.8 RNA extraction and quantitative real-time PCR

RNA extraction of ovarian tissue was performed by resuspending 50 mg of frozen tissue in 1 mL Trizol (Life Technologies, United States) and milling using a tissue lyser (Qiagen, Germany). The total RNA was isolated as described by the Trizol manufacturer. The purity and concentration of high-quality total RNA was determined by Tecan infinite M200 Pro (Grdig, Austria). cDNA was synthesized from 1 μg total RNA using the RevertAid First Chain cDNA Synthesis Kit (Thermo Fisher, United States). Real-time qPCR was performed using a 2 × Real star green fast blend (GenStar, Beijing, China). All data were normalized to *β-actin* level and analyzed according to the 2^–ΔΔ Ct^ method. The primer sequences used are listed in Supplementary Table S1.

### 2.9 Statistical analysis

All results were analyzed by GraphPad Prism 9 and expressed as the mean ± standard deviation (SD). The Student’s *t*-test was used to estimate the significance for two groups. Besides, the spearman and pearson correlation assay were conducted by the OmicShare tools, a free online platform for data analysis (http://www.omicshare.com/tools). Significance was considered at a *p*-value < 0.05.

## 3 Results

### 3.1 The differences in laying performance between YS and GYR chickens

**FIGURE 2:**
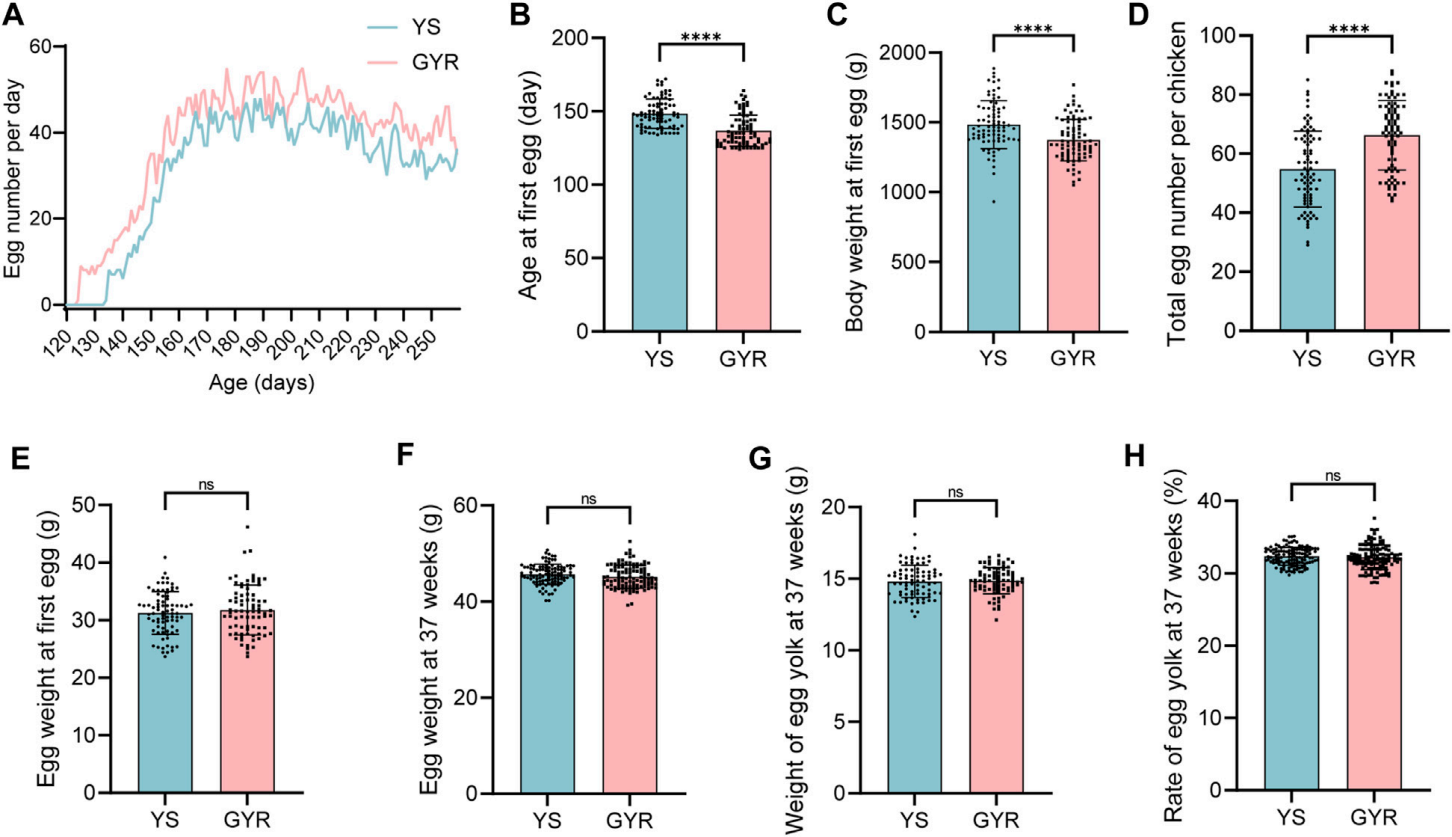
Effect of cross breeding on egg production. **(A)** The egg number for 80 layers per day. **(B)** the age at first egg (*n* = 80). **(C)** The body weight at first egg (*n* = 80). **(D)** The total egg number per layer (*n* = 80). **(E)** The egg weight at first egg (*n* = 80). **(F)** The egg weight at 37 weeks (*n* = 80). **(G)** The weight of egg yolk at 37 weeks (*n* = 80). **(H)** The rate of egg yolk at 37 weeks (*n* = 80). All the results are shown as the means ± SDs; ns represents not significant; **** represents *p*-value < 0.0001.

After 37 weeks of standardized feeding of YS and GYR chickens, the production data were analyzed to explore the differences in laying performance (Table 2). Figure 2A shows the number of eggs laid per day of YS and GYR chickens from the first laying to the end of the experiment. Besides, the age of GYR chickens at first egg laying was significantly earlier than that of YS chickens (Figure 2B). The body weight at first egg of GYR chickens is lower than that of YS chickens (Figure 2C). Most importantly, compared with the YS group, the total egg number per chicken in the GYR group significantly increased (Figure 2D). There was no significant difference in egg weight, yolk weight and yolk percentage among the three indexes related to egg quality (Figures 2E–H). Therefore, these results reveal that GYR chicken has superior reproductive performance to YS chicken.

**TABLE 2:** The descriptive statistics of egg laying performance for YS and GYR.

Traits	*n*	YS		GYR		*p*-value
		Mean ± SD	CV (%)	Mean ± SD	CV (%)	
AFE (day)	80	148.45 ± 10.04	6.77	136.83 ± 10.59	7.74	<0.0001
BWF (g)	80	1,484.57 ± 171.32	11.54	1,373.63 ± 149.50	10.88	<0.0001
TEN	80	54.83 ± 12.83	23.40	66.25 ± 11.81	17.83	<0.0001
EWF (g)	80	31.28 ± 3.72	11.90	31.77 ± 4.33	13.64	0.443
EW37 (g)	80	45.70 ± 2.03	4.44	45.14 ± 2.43	5.38	0.110
WEY37 (g)	80	14.81 ± 1.13	7.63	14.86 ± 0.91	6.12	0.802
REY37 (%)	80	32.48 ± 2.94	4.63	33.00 ± 2.58	7.82	0.243

AFE, the age at first egg; BWF, the body weight at first egg; TEN, the total egg number for 37 weeks per layer; EWF, the egg weight at first egg; EW37, the egg weight at 37 weeks; WEY37, the weight of egg yolk at 37 weeks; REY37, the rate of egg yolk at 37 weeks; SD, standard deviation; CV, the coefficient of variance.

### 3.2 Overview of RNA sequencing and quality control

**FIGURE 3:**
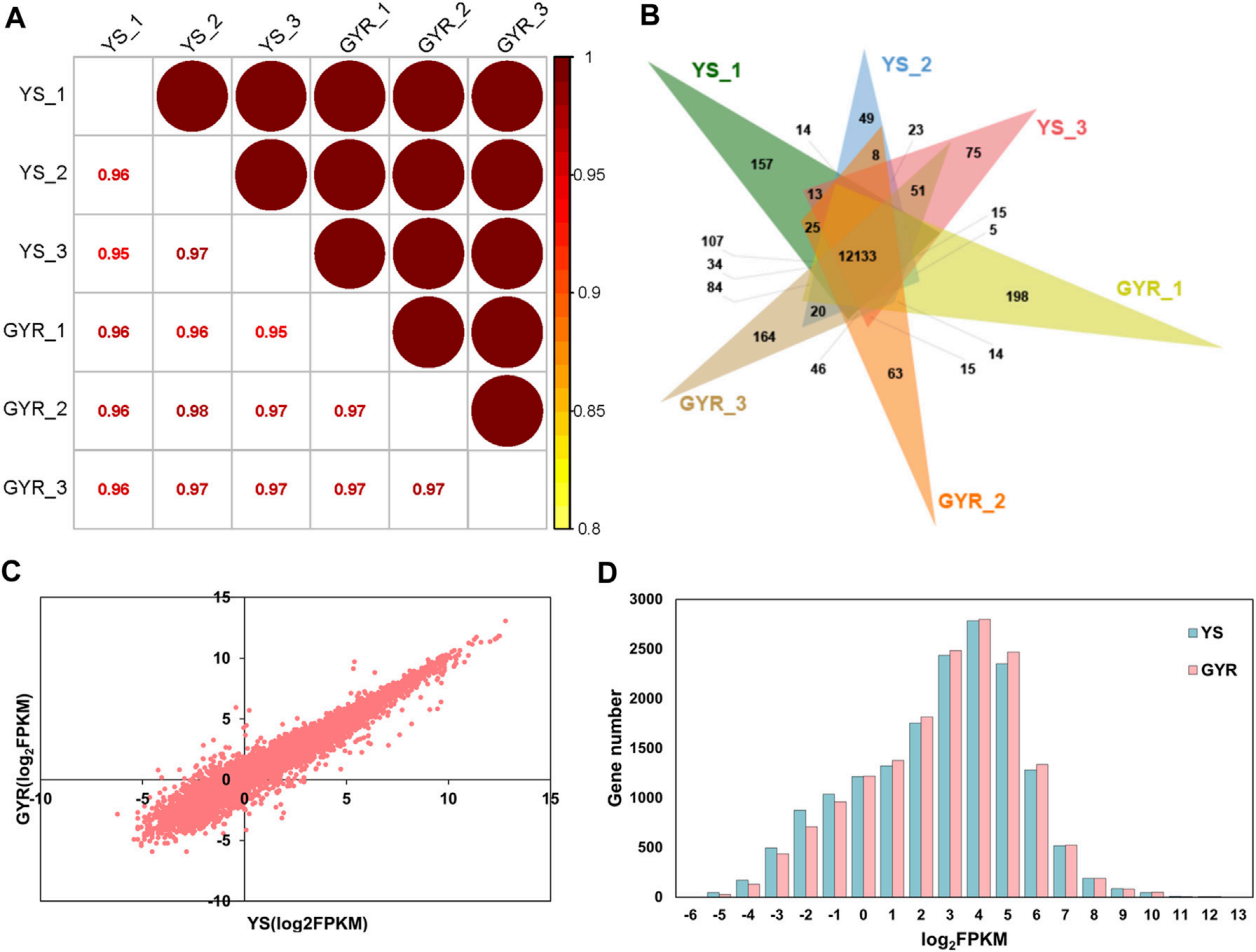
Characteristics of ovarian transcriptome. **(A)** Spearman correlation analysis of gene expression abundance between YS and GYR. **(B)** The petal Venn diagram of genes identified by RNA-seq. **(C)** Expression abundance correlation analysis between YS and GYR in RNA-seq. **(D)** Distribution of the mRNA abundance.

To identify the molecular regulatory mechanism of laying performance in YS and GYR chickens, RNA-seq was performed on the ovarian tissues of these chickens (each with three biological replicates). In total, 44.2–46.2 million and 44.4–55.8 million clean reads were generated for YS and GYR chickens, respectively, and the Q30 of all samples was greater than 92% (Table 3). The identification and quantification information of the transcriptome is shown in Supplementary Table S2. Spearman correlation analysis showed a high correlation (*R* ^2^ > 0.95) between the RNA-seq biological replications (Figure 3A). The petal Venn diagram shows that 12,133 genes were identified in all ovarian tissue samples (Figure 3B), and there was a strong correlation between YS and GYR chickens (*R* ^2^ = 0.9538; Figure 3C). The abundance distributions of mRNA in YS and GYR chickens were approximately lognormal (μ = 4; Figure 3D). These results indicate that these data are reliable and can be used for subsequent analyses.

**TABLE 3:** Sequencing data statistics and sequencing quality assessment.

Sample ID	Raw reads	Clean reads	Q20 (%)	Q30 (%)	GC rate (%)
YS_1	46,314,258	44,207,684	97.03	92.20	52.48
YS_2	47,213,772	45,296,974	97.13	92.38	51.44
YS_3	48,873,440	46,217,634	97.06	92.29	51.96
GYR_1	50,394,464	47,644,980	97.06	92.27	52.20
GYR_2	58,519,026	55,807,988	97.67	93.62	51.28
GYR_3	46,877,498	44,420,370	97.03	92.21	52.33

### 3.3 Differential transcriptome analysis

**FIGURE 4:**
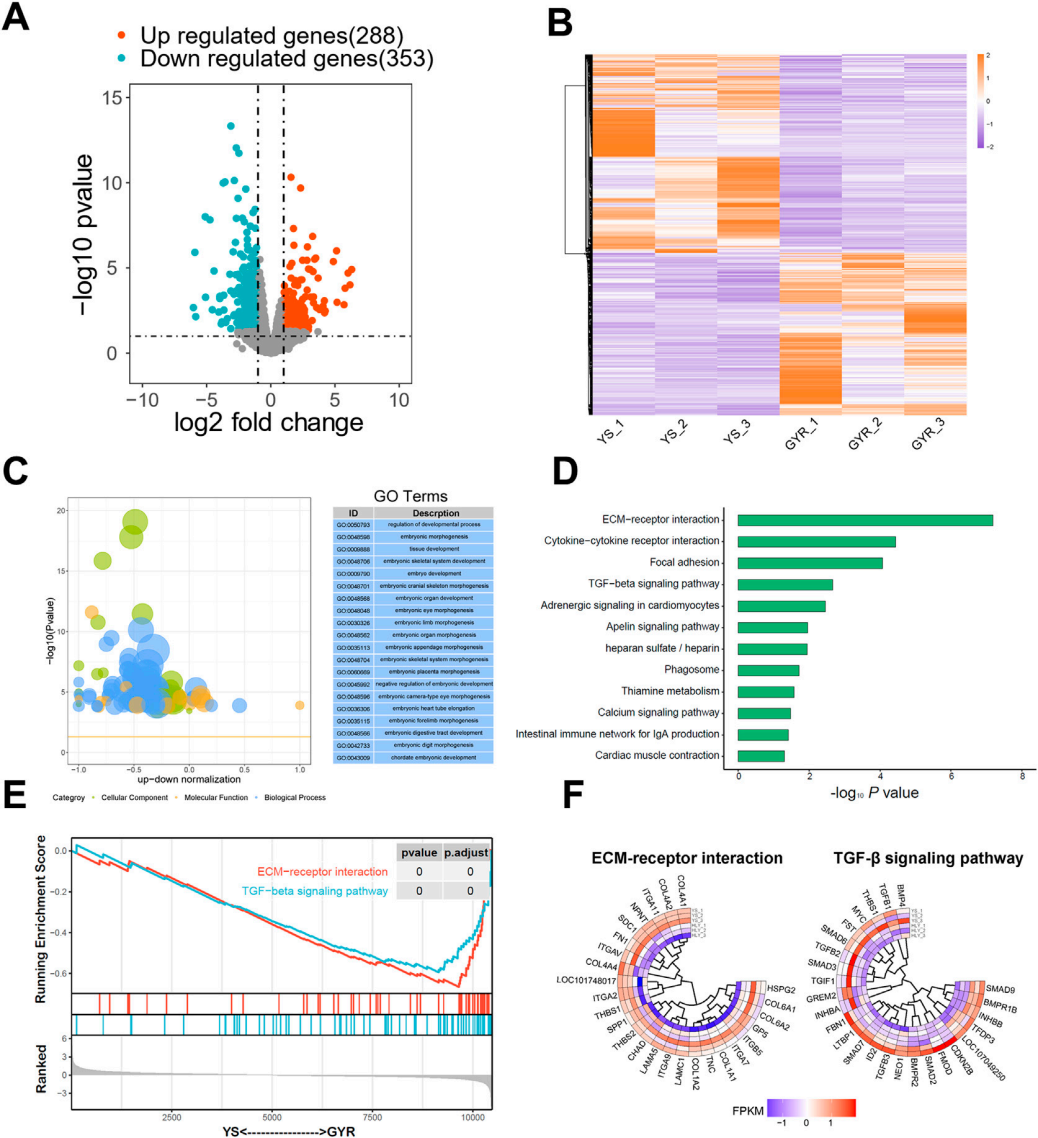
Identification of DEGs and functional enrichments. **(A)** The volcano map of DEGs. The upregulated genes with *p*-value < 0.05 and log_2_ Fold Change >1 are marked in red; the downregulated genes with *p*-value < 0.05 and log_2_ Fold Change < −1 are marked in blue. **(B)** YS and GYR ovarian can be clearly distinguished based on their transcriptome characteristics. The color key (from purple to red) of abundance value indicated low to high expression levels. **(C)** GO analysis of DEGs. **(D)** KEGG analysis of DEGs. **(E)** GSEA-KEGG analysis of the transcriptome. A pathway of positive enrichment score is upregulated, whereas a pathway of negative enrichment score is downregulated. **(F)** Gene expression heatmap of the ECM-receptor interaction and TGF-β signaling pathway. The color key (from purple to red) of abundance value indicated low to high expression levels.

To compare the transcriptional profiles of YS and GYR chicken ovaries, we used the DEseq2 R package to screen DEGs. Using |log_2_ fold change| > 1 and *p*-value < 0.05 as the screening criteria, 641 DEGs were identified, among which 353 DEGs were upregulated in YS chickens and 288 DEGs were upregulated in GYR chickens (Figure 4A; Supplementary Table S3). Cluster analysis of the DEGs revealed that the gene expression patterns were clustered within groups and the difference was significant between groups (Figure 4B). In summary, we found significantly differentially expressed genes in the ovarian tissues between YS and GYR chickens.

To elucidate the contribution of the specific signaling pathways of the DEGs found between YS and GYR chicken ovaries, GO and KEGG functional enrichment analyses were conducted. The GO terms were classified into three categories: biological process, molecular function and cellular component. Here, we mainly focused on the differences in egg production between YS and GYR chickens during peak laying periods. The GO enrichment results revealed that more than 20 terms were involved in reproductive regulation, including regulation of developmental process, embryonic morphogenesis, tissue development, embryonic skeletal system development and so on (Figure 4C; Supplementary Table S4). The KEGG enrichment analysis identified that the DEGs were significantly enriched in certain pathways, including ECM-receptor interaction and TGF-β signaling pathway (Figure 4D). GSEA further revealed that the ECM-receptor interaction pathway and TGF-β signaling pathway were significantly downregulated in GYR chickens (Figure 4E). Figure 4F presents the gene set and expression abundance of the ECM-receptor interaction pathway and TGF-β signaling pathway.

### 3.4 Metabolic profiling of chicken ovaries

**FIGURE 5:**
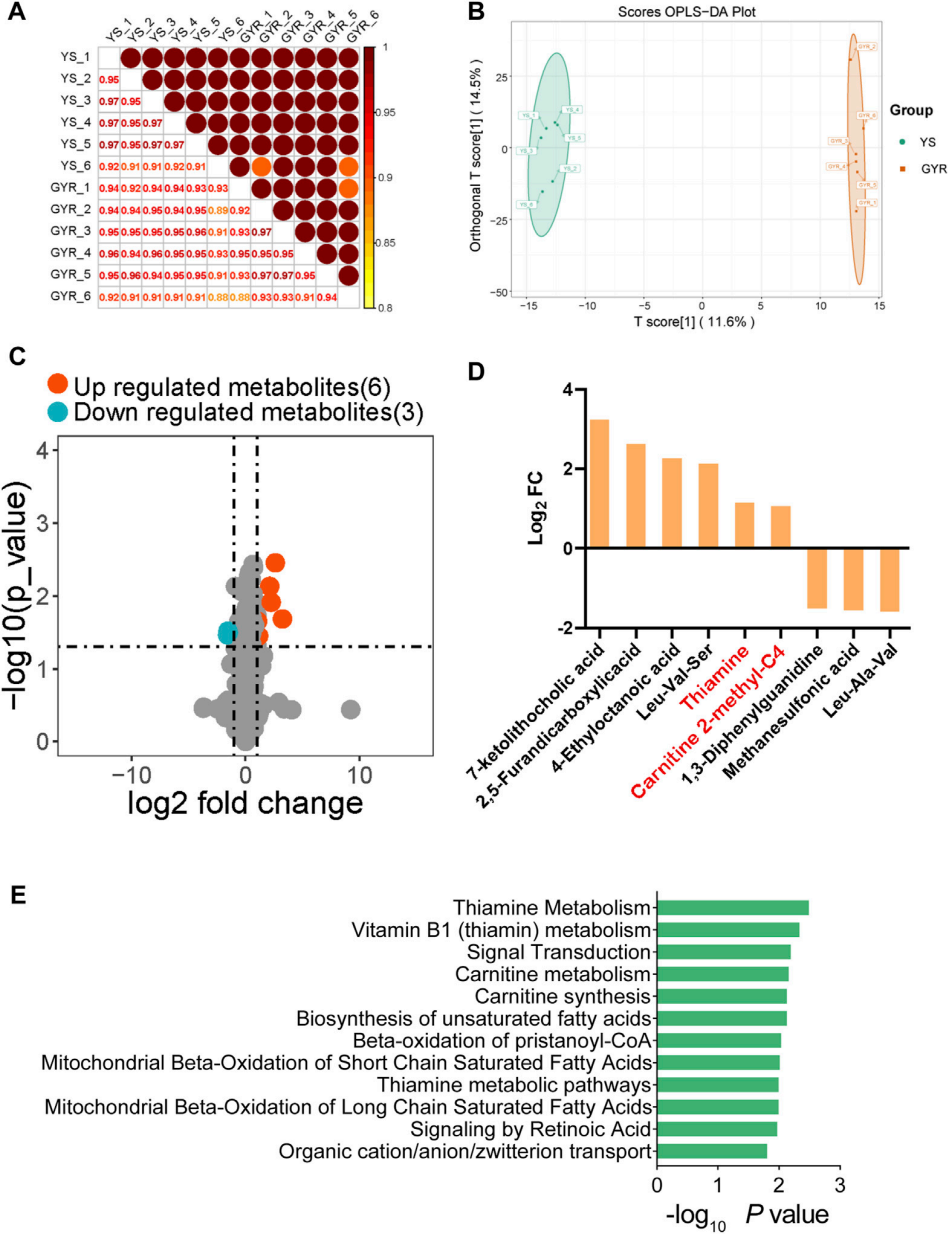
Analysis of ovarian metabolomics. **(A)** Spearman correlation of metabolic expression between YS and GYR. **(B)** OPLS-DA score plot of all groups. **(C)** The volcano map of SDMs. The upregulated metabolisms with VIP >1, log_2_ fold change >1 and *p*-value < 0.05 are marked in red; the downregulated metabolisms with VIP >1, log_2_ fold change < −1 and *p*-value < 0.05 are marked in blue. **(D)** The fold change of SDMs. **(E)** The KEGG functional enrichment analysis of SDMs.

Using the UPLC‒MS/MS platform of the untargeted metabolome, we annotated metabolites from the ovaries from YS and GYR chickens (each with six biological replicates), and positive ion and negative ion modes were used. Analyst 1.6.3 software was used for quality control of the extracted substances. The total ion current (TIC) of the QC samples was compared with the overlapping spectra. The response intensity and retention time of each chromatographic peak overlapped, indicating that there was little variation caused by instrument error throughout the whole experiment, and the data quality was reliable (Supplementary Figures S1A, B). A total of 1,631 metabolites were identified in the metabolome (Supplementary Table S5). Spearman correlation analysis showed high similarity between biological replicates (*R* ^2^ > 0.88), which was in line with our expectation (Figure 5A). Orthogonal partial least squares discriminant analysis (OPLS-DA) showed that the differences within groups were small, but the differences between the YS and GYR chickens were significant, and the two chicken breeds were well differentiated (Figure 5B).

To investigate the metabolic differences between YS and GYR chickens, significantly differential metabolites (SDMs) were identified. According to the thresholds |log_2_ fold change|>1, *p*-value < 0.05 and VIP > 1, we screened 9 SDMs (6 upregulated and 3 downregulated metabolites; Figure 5C; Supplementary Table S6). Figure 5D displays the SDMs, including 7-ketolithocholic acid, 2,5-furandicarboxylic acid, 4-ethyloctanoic acid, leu-val-ser, thiamine, carnitine 2-methyl-C4, 1,3-diphenylguanidine, methane sulfonic acid and leu-ala-val. Figure 5E shows the enrichment analysis of differential metabolites.

### 3.5 Combined analysis of the transcriptome and metabolome of chicken ovaries

**FIGURE 6:**
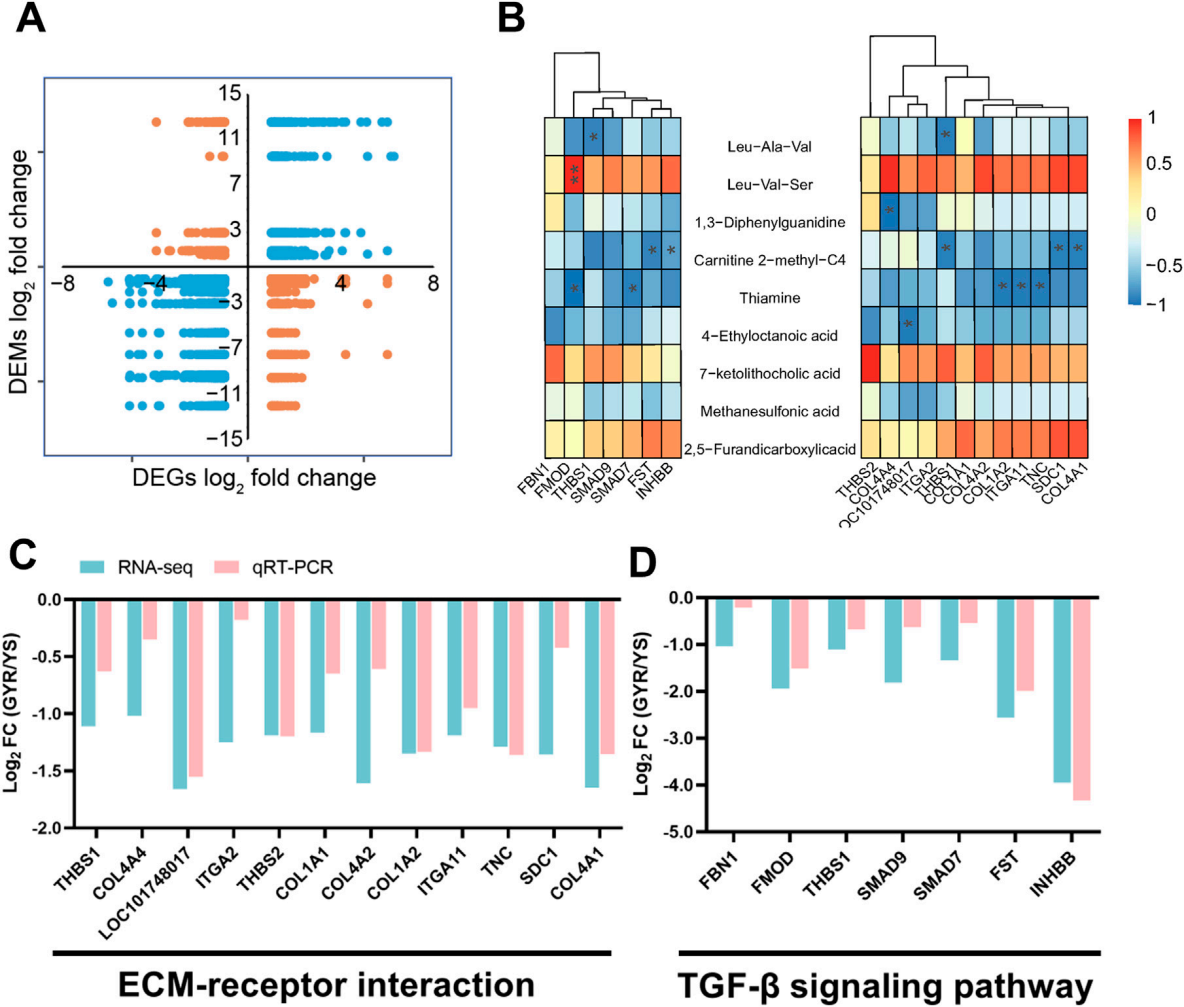
Correlation analysis and quantitative verification. **(A)** The nine-quadrant plot between differential genes and metabolites. **(B)** Combine analysis of SDMs and some genes in ECM-receptor interaction and TGF-β signaling pathway. **(C,D)** qRT-PCR analysis of the genes of ECM-receptor interaction and TGF-β signaling pathway. All the results are shown as the means ± SD; * represents *p*-value < 0.05; ** represents *p*-value < 0.01.

The above analyses delineated the transcriptional and metabolic profiles of Yaoshan chickens and GYR chickens with higher egg production performance. For transcriptome analysis, 641 DEGs were identified (Figure 4A). In addition, 9 SDMs were screened from the metabolome (Figure 5D). The nine-quadrant plot was utilized to visually represent correlation analyses between differential genes and metabolites (Figure 6A; Supplementary Table S7). To further narrow the focus, we found that ECM-receptor interactions and the TGF-β signaling pathway were significantly inhibited in the transcriptome enrichment analyses (Figure 4E). Integrated analysis of transcriptome and metabolites in three paired samples validated the association of ECM-receptor interactions and the TGF-β signaling pathway with SDMs. Figure 6B analyses the correlation between the SDMs and DEGs in terms of ECM-receptor interactions and the TGF-beta signaling pathway. Next, the gene expression of the two pathways was verified quantitatively by qRT-PCR, and the results were consistent with transcriptome sequencing (Figures 6C, D). The results showed that thiamine and carnitine were negatively correlated with the DEGs involved in ECM-receptor interactions and the TGF-beta signaling pathway.

## 4 Discussion

Egg production is an important economic indicator in chickens and is closely related to the benefits for agricultural enterprises. The majority of the indigenous chicken breeds in China are poor at laying eggs compared with commercial layers (Zhao et al., 2018; Guo et al., 2021). Therefore, improving the laying performance of local chicken breeds has always been one of the main purposes of breeding. Crossbreeding is an important method that is widely used in the production of commercial chickens (Soliman et al., 2020). In this study, high-yielding recessive white feather layers (RW) and green-shell chickens with yellow feathers (GY) were utilized to crossbreed and improve YS hens. We measured the laying performance of commercial chickens (GYR) and YS chickens, and the results showed that GYR chickens displayed good heterosis in terms of the age at first egg and egg production (Figure 2F).

### 4.1 Transcriptomics analysis

In this study, we identified 641 DEGs in the ovaries of two laying hens (GYR vs. YS), with 288 upregulated and 353 downregulated genes. These results suggested that these DEGs might play an important role in the regulation of egg production. The results of GO enrichment analysis showed that these DEGs were mainly enriched in related embryonic development terms, such as embryonic morphogenesis and embryonic development. The GSEA-KEGG results showed that ECM-receptor interactions and the TGF-β signaling pathway were inhibited in GYR chicken ovaries. Previous studies have shown that TGF-β inhibitors can improve excessive ovarian fibrosis in model rats and promote ovarian function (Wang et al., 2018). In addition, TGF-β1 and TGF-β2 reduce somatic cell numbers in chick embryo ovaries by inhibiting somatic cell proliferation without increasing apoptosis (Mendez et al., 2006). As in GYR laying hens, downregulation of the TGF-β signaling pathway can reduce ovarian maturation time and increase egg production. It has also been reported by others that ECM-receptor interactions are related to ovarian maturation at different stages in laying hens and the deceleration and division of oocytes (Dong et al., 2021). Additionally, knockdown of TIMP3 reduced hCG-induced progesterone secretion and the mRNA abundance of key steroidogenic enzymes and ECM proteins, decreased apoptosis and increased granulosa cell viability (Peng et al., 2015). In brief, the TGF-β signaling pathway and ECM-receptor interactions are considered to be closely related to the egg-laying performance of chickens.

TGF-β family members are important paracrine and autocrine signaling molecules, which dynamically regulate the physiological function of ovary (Lu et al., 2022). Follistatin (FST), inhibin β B subunit (INHBB) and fibromodulin (FMOD) are involved in the TGF-β signaling pathway. FST is a single-chain gonadal protein that specifically inhibits the release of follicle-stimulating hormone (FSH), boosting the development and reproduction of animals (Shi et al., 2016). Evidence shows that eliminating the biological activity of FST can promote the growth and differentiation of goose precursor follicles by enhancing SMAD3 (Chen et al., 2022). INHBB is a glycoprotein hormone that inhibits the production and secretion of FSH (Yuan et al., 2020). INHBB gene knockout can promote granulosa cell apoptosis and inhibit steroid production, which is closely related to the reproductive process in sheep (M'Baye et al., 2015). Consistent with our results, low expression of the INHBB transcript in the ovaries of GYR hens can promote hormone secretion and increase egg production. FMOD is a member of the family of small gap proteoglycans that affects the rate of fibril formation (Han et al., 2022; Yang et al., 2022). Previous studies have shown that in bovine ovarian cells, normal developing oocytes show low FMOD expression (Martinez-Moro et al., 2022). This is consistent with our study, which also showed low FMOD mRNA expression in the ovaries of high-yielding GYR hens. Additionally, heparan sulfate proteoglycan 2 (HSPG2) participates in ECM-receptor interactions. Previous conclusions have suggested that low HSPG2 expression is associated with more mature oocytes, transferable embryos, and high-quality embryos (Ma et al., 2020). In summary, one of the reasons for the increase in egg production from high-yield GYR laying hens after hybridization is inhibition of the TGF-β signaling pathway, which promotes ovarian development.

### 4.2 Metabolomics analysis

In this study, we found 9 SDMs in the ovaries of chickens with different egg laying rates from our self-built database, of which 6 SDMs were higher and 3 SDMs were lower in GYR chickens. Among these SDMs, thiamine and carnitine metabolites have been reported to be associated with egg production and ovarian development. Thiamine, also known as vitamin B-1, is associated with cell macromolecular structure maintenance, cell growth and proliferation, oxidative stress prevention and so on (Frank, 2015). Thiamine can prevent and repair ischaemia/reperfusion injury and rat ovarian tissue infertility and restore ovarian function (Demiryilmaz et al., 2013). This is consistent with our findings that compared with YS hens, GYR hens have more thiamine in their ovaries, which is helpful for ovarian function. The maturation of follicles and meiosis of oocytes require considerable energy (Warzych and Lipinska, 2020). Carnitine, an endogenous molecule, transports fatty acid chains into the mitochondrial matrix, allowing cells to breakdown fat and obtain energy from stored fat reserves (Pekala et al., 2011). In previous studies, carnitine increased the beta-oxidation of lipids in mouse follicles to provide energy for cell division (Dunning et al., 2011). Similarly, in laying hens and pigs, supplementation with carnitine can increase egg production and improve reproductive performance (Rabie et al., 1997; Wu et al., 2011). This is also highly consistent with the phenotype of GYR laying hens, so it is likely a direct factor that affects the high egg production of GYR laying hens.

## 5 Conclusion

In summary, this study shows that, after analysis at the transcriptional level, the higher egg production of the GYR layer compared with that of the YS layer at might be due to inhibition of the TGF-β signaling pathway and the ECM-receptor interactions. Metabonomic research has shown that increases in carnitine and thiamine enhance energy metabolism and antioxidant performance, which may be a direct factor affecting egg production. These results are also helpful to explain the different molecular mechanism of egg production between GYR and YS chickens. It is worth noting that there are some strength and weakness in this study. Yaoshan chickens and GYR chickens are less researched breeds, and investigating their egg-laying performance significantly contributes to the accumulation of valuable genetic resources. Additionally, the transcriptomic and metabolomic analyses employed in this study offer valuable insights for identifying potential genes and metabolites associated with production performance. However, applying these findings to practical production scenarios remains a challenge, which will be the focus of our future work.

## Data Availability

The data presented in the study are deposited in the GEO repository, accession number is GSE218287.

## Supplementary material

SUPPLEMENTARY FIGURE S1 The total ion chromatogram (TIC) of metabolites. **(A,B)** Total positive ions and negative ions UPLC-MS/MS chromatograms of ovary sample with the differential metabolites labeled.

SUPPLEMENTARY TABLE S1 Primers used for a quantitative polymerase chain reaction.

SUPPLEMENTARY TABLE S2 Gene expression profile.

SUPPLEMENTARY TABLE S3 The information of DEGs.

SUPPLEMENTARY TABLE S4 Summary of the GO results.

SUPPLEMENTARY TABLE S5 Metabolite expression profile.

SUPPLEMENTARY TABLE S6 The information of SDMs.

SUPPLEMENTARY TABLE S7 The nine-quadrant plot of DEGs and SDMs.
