# Supplementary material for: Integrative analysis of the ovarian metabolome and transcriptome of the Yaoshan chicken and its improved hybrids
Source: Front Genet. 2024 Jul 8;15:1416283. doi: 10.3389/fgene.2024.1416283 (PMC11260793; doi:10.3389/fgene.2024.1416283)
Supplement: Supplementary file 3 [file Table1.DOCX]

**Supplemental Table 1.** Primers used for a quantitative polymerase chain reaction.

| **Gene name** | **Accession number** | **Primer (5’-3’)** | **Product size, bp** |
| --- | --- | --- | --- |
| β-actin | NM_205518.2 | F: TGCGTGACATCAAGGAGAAG | 300 |
|  |  | R: TGCCAGGGTACATTGTGGTA |  |
| THBS1 | NM_001199453.2 | F: ACCAACCAAAGCTCAAGGCT | 131 |
|  |  | R: GCCACAAAGTTCTCACCTGG |  |
| COL4A4 | XM_015276987.4 | F: GACGTGGGGTCAGTTGGAC | 80 |
|  |  | R: AGGCCAGGCAGACTTGTTC |  |
| LOC101748017 | XM_025143528.3 | F: TCGGCGTATCAGTTCCACAG | 217 |
|  |  | R: TGATGTGGGTCACCGAGAAG |  |
| ITGA2 | XM_003642982.6 | F: TTCCTCCTTACCGAGCGATG | 110 |
|  |  | R: ACACCTGTTGTCCTGTTCGG |  |
| COL1A1 | NM_001396622.1 | F: CGACGGCTTCCAGTTTGAGT | 119 |
|  |  | R: TGCAGTGGTAGGTGACGTTC |  |
| THBS2 | NM_001397325.1 | F: GGGATTGCCCAATTGATGGT | 229 |
|  |  | R: CATGGCAAGCAGTGGAAACC |  |
| COL1A2 | NM_001079714.2 | F: GAAGATGGTCACCCTGGCAA | 131 |
|  |  | R: TCCAGACCATTGTGTCCCCTA |  |
| COL4A2 | NM_001162390.2 | F: CAGATGGCGTTCCTGGTCAT | 298 |
|  |  | R: CTTCCTGGCACTCCTCTTGG |  |
| ITGA11 | XM_040706686.2 | F: TGTGACAGTCATGCAGAGCC | 154 |
|  |  | R: CCCACCACCAGCCATTTCTT |  |
| TNC | NM_205456.5 | F: CGGCTACAACAGAGGCAGAA | 95 |
|  |  | R: CCCATCATCTGCAGTCCAGG |  |
| COL4A1 | NM_001162399.4 | F: ATCTAGGGCCTCCAGGTGTT | 246 |
|  |  | R: GCCCTGTTACTCCTTGCTGT |  |
| SDC1 | XM_419972.7 | F: TCGAACCTAAAACCCCTGGC | 310 |
|  |  | R: CCTCCAGCAATAACACCTCCA |  |
| FBN1 | XM_015291934.4 | F: AGGAAGCGGATGAGGCATTC | 75 |
|  |  | R: GTCTTGCATCACCAGGTCCA |  |
| FMOD | NM_204967.2 | F: GGCTCTCTGCTTGGTCCCTC | 131 |
|  |  | R: ATGTAGTACTGCAGCCACGC |  |
| SMAD7 | XM_427238.8 | F: CGAGCTCGAGTCTCCTCCT | 76 |
|  |  | R: GTCTGGACAACCTGCCGTC |  |
| SMAD9 | NM_001024826.1 | F: AAACAGGTTCTGCCTGGGAC | 350 |
|  |  | R: CTGCTCCCCAGCCTTTAACA |  |
| INHBB | XM_025152149.3 | F: AAGCAACCCGACGGGGA | 189 |
|  |  | R: TGTTTGATCTCCGCTCGCC |  |
| FST | NM_205200.2 | F: ACACAGCGCAGGCTGG | 146 |
|  |  | R: GTTGTCGTTGACGTCCTCCT |  |
